# Endothelium-targeted delivery of dexamethasone by anti-VCAM-1 SAINT-O-Somes in mouse endotoxemia

**DOI:** 10.1371/journal.pone.0196976

**Published:** 2018-05-15

**Authors:** Ranran Li, Piotr S. Kowalski, Henriëtte W. M. Morselt, Ilona Schepel, Rianne M. Jongman, Adnan Aslan, Marcel H. J. Ruiters, Jan G. Zijlstra, Grietje Molema, Matijs van Meurs, Jan A. A. M. Kamps

**Affiliations:** 1 Dept. of Pathology & Medical Biology, Medical Biology Section, Laboratory for Endothelial Biomedicine & Vascular Drug Targeting Research, University Medical Center Groningen, University of Groningen, Groningen, The Netherlands; 2 Dept. of Critical Care, University Medical Center Groningen, University of Groningen, Groningen, The Netherlands; 3 Dept. of Anesthesiology, University Medical Center Groningen, University of Groningen, Groningen, The Netherlands; Monash University, AUSTRALIA

## Abstract

Microvascular endothelial cells play a pivotal role in the pathogenesis of sepsis-induced inflammatory responses and multiple organ failure. Therefore, they represent an important target for pharmacological intervention in the treatment of sepsis. Glucocorticosteroids were widely used in the treatment of sepsis but vast evidence to support their systemic use is lacking. The limited effects of glucocorticoids in the treatment of sepsis may be explained by differential effects of drug initiated NF-κB inhibition in different cell types and insufficient drug delivery in target cells. The current study aimed therefore to investigate the effects of an endothelial targeted delivery of dexamethasone in a mouse model of endotoxemia induced by two consecutive i.p. injections of lipopolysaccharide (LPS). To achieve endothelial cell specific delivery of dexamethasone, we modified SAINT-O-Somes, a new generation of liposomes that contain the cationic amphiphile SAINT-C18 (1-methyl-4-(cis-9-dioleyl) methyl-pyridinium chloride, with antibodies against vascular cell adhesion molecule-1 (VCAM-1). In LPS challenged mice, the systemic administration of free dexamethasone had negligible effects on the microvascular inflammatory endothelial responses. Dexamethasone-loaded anti-VCAM-1 SAINT-O-Somes specifically localized at VCAM-1 expressing endothelial cells in the microvasculature of inflamed organs. This was associated with a marginal attenuation of the expression of a few pro-inflammatory genes in kidney and liver, while no effects in the lung were observed. This study reveals that, although local accumulation of the targeted drug was achieved, endothelial targeted dexamethasone containing anti-VCAM-1 SAINT-O-Somes exhibited marginal effects on inflammatory endothelial cell activation in a model of endotoxemia. Studies with more potent drugs encapsulated into anti-VCAM-1 SAINT-O-Somes will in the future reveal whether this delivery system can be further developed for efficacious endothelial directed delivery of drugs in the treatment of sepsis.

## Introduction

Sepsis is characterized by uncontrolled systemic inflammation triggered by an infection, and is the most common cause of death among hospitalized patients. Despite substantial efforts in understanding the pathophysiology of sepsis and investigating potential therapeutic strategies, effective treatment of sepsis remains a clinical challenge [1].

The pathophysiology of sepsis is complex and multifactorial, one of the key deteriorations is microvascular leakage and microvascular inflammation. Endothelial cells (EC) play a central role in regulating the processes of vascular leakage and inflammation. By expressing and releasing adhesion molecules, cytokines, and chemokines endothelial cells orchestrate the recruitment of leukocytes from the blood into underlying tissue and changes in vascular barrier function [2]. In sepsis induced multiple organ dysfunction syndrome (MODS), this process is dysregulated resulting in uncontrolled inflammation and massive (vascular) leakage. The accessibility of EC for intravenously administered substances and their heterogeneity in behavior allow for organ microvascular and/or disease specific drug delivery. Upon inflammatory stimulation, a vascular bed specific expression pattern of cell adhesion molecules such as E-selectin and vascular cell adhesion molecule (VCAM)-1 is induced [3], providing opportunities for specific delivery of therapeutic reagents to diseased (micro)vascular endothelial subsets [4,5].

Glucocorticoids (GC) were widely used in the treatment of sepsis patients because they are thought to diminish systemic and tissue inflammation and restore organ functions [6,7], but vast evidence to support their use is lacking [8]. Glucocorticoids exert their effects by binding to intracellular glucocorticoid receptors (GRs) in the cytoplasm which then translocate into the nucleus. There the GR complex regulates inflammatory responses through transactivation of anti-inflammatory genes and the inhibition of nuclear factor kappa B (NF-κB) and activator protein (AP)-1 driven inflammatory mediators such as cytokines and adhesion molecules [9]. The limited effects of systemic administration of glucocorticoids in the treatment of sepsis may be explained by differential effects of drug-initiated NF-κB inhibition in different cell types and possible low concentrations in target cells in sepsis. It has been shown that in a rodent sepsis model specific endothelial NF-κB inhibition is protective for the host [10] while for white blood cell NF-κB inhibition was detrimental [11]. Therefore, a strategy where glucocorticoids are specifically delivered to inflamed endothelial cells might overcome these opposing effects in favour of the protective action of GC.

Selective delivery of therapeutic molecules can be achieved using liposomes that are designed to encapsulate pharmacologically active entities. When liposomes are modified with monoclonal antibodies, they become drug carriers with binding specificity for selective epitopes [12]. We have previously demonstrated that endothelial specific delivery of liposome-encapsulated dexamethasone attenuates the expression of pro-inflammatory genes in a murine model of glomerulonephritis. By delivering the drugs locally, an increase in systemic glucose levels as one of the side effects of systemic administration of dexamethasone was effectively avoided [13]. To create superior intracellular release of their content in endothelial cells compared to conventional liposome technology, we have designed a new generation of liposomes, by combining the cationic lipid SAINT-C18 (1-methyl-4-(cis-9-dioleyl) methyl-pyridinium chloride) with conventional liposome formulation [14]. In previous studies we showed that these so-called SAINT-O-Somes, when targeted to E-selectin and VCAM-1, can effectively deliver small interfering RNA into inflamed endothelial cells both *in vitro* and *in vivo* [15,16].

In the current study, we aimed to evaluate the targeting specificity of dexamethasone loaded anti-VCAM-1 (Ab_VCAM-1_) SAINT-O-Somes to inflamed endothelium in an endotoxemia mouse model as well as their effects on microvascular endothelial activation *in vivo*. We used an LPS model in which animals were challenged with two consecutive i.p. injections of 1 mg/kg LPS. We investigated the association of Ab_VCAM-1_ SAINT-O-Somes with LPS activated endothelial cells *in vitro* and their accumulation at the site of inflamed microvasculature in 3 organs that are affected by sepsis/endotoxemia challenge *in vivo*, and studied the pharmacological effects of endothelial targeted dexamethasone in the microvasculature in the organs.

## Materials and methods

### Materials

#### Lipids

Cholesterol (Chol) was obtained from Sigma (St. Louis MO, USA). 1-methyl-4-(cis-9-dioleyl) methyl-pyridinium-chloride (SAINT-C18) was obtained from Synvolux Therapeutics Inc. (Groningen, The Netherlands). 1-palmitoyl-2-oleoyl-sn-glycero-3-phosphocholine (POPC), 2-distearoyl-sn-glycero-3-phosphoethanolamine-N- [methoxy(polyethyleneglycol)-2000] (DSPE-PEG2000) and 1,2-distearoyl-sn-glycero-3-phosphoethanolamine-N-[methoxy(polyethyleneglycol)-2000]-maleimide (Mal-PEG2000 -DSPE) were obtained from Avanti Polar Lipids (Alabaster, AL, USA).

#### Antibodies

E1/6-aa2 (mouse IgG1 anti-human VCAM-1 antibody) monoclonal antibody-producing hybridoma were kindly provided by Dr. M. Gimbrone from Harvard Medical School (Boston, MA, USA). The M/K-2.7 (rat IgG1a anti-mouse VCAM-1 antibody) producing hybridoma was obtained from American Type Culture Collection (ATCC, Manassas, VA, USA). Rat IgG antibody (used as irrelevant IgG) was purchased from Sigma-Aldrich (Zwijndrecht, The Netherlands).

#### Other reagents

N-succinimidyl-S-acetylthioacetate (SATA) was obtained from Sigma (St. Louis, MO, USA). Nucleic acid stain Hoechst 33342 (trihydrochloride) and lipophilic tracer 1,1′-dioctadecyl-3,3,3′,3′-tetramethyl-indocarbocyanine perchlorate (DiI) were obtained from Molecular Probes (Leiden, The Netherlands).

### Preparation and characterization of SAINT-O-Somes and liposomes

For the preparation of SAINT-O-Somes, lipids from stock solutions in chloroform: methanol (9 : 1), i.c., Chol, POPC, SAINT-C18, DSPE-PEG2000 and Mal-PEG2000-DSPE, were mixed in a mol% ratio of 40 : 37 : 18 : 4 : 1. To label the SAINT-O-Somes fluorescently, DiI was added to the lipid mixture in a 0.25 mol% ratio of total lipid (TL). The lipids were dried under a stream of nitrogen. To form dexamethasone containing SAINT-O-Somes, the lipids were hydrated in 145 mM dexamethasone disodium phosphate (Pharmacy UMCG, Groningen, The Netherlands). To form control SAINT-O-Somes without dexamethasone, the lipids were hydrated in HN buffer (135 mM NaCl, 10 mM HEPES, pH 6.7). The concentration of lipids during the hydration was 40 μmol TL/ml. After hydration, 10 cycles of rapid freezing (liquid nitrogen) and thawing (warm water, 40°C) were performed. The formed SAINT-O-Somes were sized by repeated and successive extrusion at 40°C through polycarbonate filters (Costar, Cambridge, MA, USA) with pore sizes of 200 nm, 100 nm, and 50 nm (5 times each). Free dexamethasone was removed by gel chromatography on a Sephadex G-50 column (Sigma-Aldrich, Zwijndrecht, The Netherlands) using HN buffer (pH 6.7) as eluent. SATA-modified anti-human VCAM-1, anti-mouse VCAM-1, respectively rat IgG antibodies were coupled to these SAINT-O-Somes by the sulfhydryl maleimide method as described before for albumin [17]. After extrusion, gel chromatography, and protein coupling, the diameter of the SAINT-O-Somes was measured using dynamic light scattering (DLS) in the volume weighing mode (NICOMP particle sizing systems, Santa Barbara, CA, USA). The SAINT-O-Somes were characterized by determining the amount of coupled protein using rat IgG as a standard and the total lipid concentration was determined by phosphorus assay as described before [16].

### Cell culture

Human umbilical vein endothelial cells (HUVEC) were isolated from umbilical cords and obtained from the Endothelial Cell Facility of the UMCG. Approval of the medical ethical committee was waived by The Medical Ethical Committee of the University Medical Center Groningen (UMCG) as umbilical cords are considered anonymous waste material. HUVEC were isolated from at least two umbilical cords to circumvent donor bias, and were cultured on 1% gelatin (Sigma, Zwijndrecht, The Netherlands) coated flasks with medium (RPMI 1640, Lonza, BE) supplemented with 20% heat inactivated FCS (Fetal Calf Serum; Thermo Scientific HyClone, Cramlington, UK), 2 mM Glutamine (Lonza, Breda, The Netherlands), 5 U/ml heparin (LEO-Pharma, Amsterdam, The Netherlands), 50 μg/ml endothelial cell growth factor, 100 μg/ml streptomycin (Rotexmedica GmbH, Trittau, Germany) and 100 IU/ml penicillin (Astellas Pharma, Meppel, The Netherlands).

### Analysis of SAINT-O-Some association with HUVEC by fluorescence microscopy and flow cytometry

For fluorescence microscopy, HUVEC were cultured on 8-well LabTek chamber slides (Nunc, Rochester, NY, USA). Cells were activated with two times lipopolysaccharide (LPS, 1 μg/ml, Escherichia coli, serotype 0.26 : B6l, Sigma, St. Louis, MO, USA) in medium containing 5% fetal calf serum (FCS, Sigma-Aldrich) (37°C, 5% CO2), with 3h in between. 2h after the first stimulation, Dil labeled anti-VCAM-1 respectively non-targeted SAINT-O-Somes containing dexamethasone (80 nmol TL/ml) were added to the cells for another 4h. Cell nuclei were stained using Hoechst 33342 (10 μg/ml) for the last 10 min of the incubation. At the end of the incubation cells were washed twice with ice-cold serum-free culture medium, placed on ice, and subjected to imaging within 45 min. Fluorescence images of cells were taken with a Leica DM/RXA fluorescence microscope using Quantimet HR600 image analysis software (Leica, Wetzlar, Germany). Images were taken at excitation/emission wavelengths of 550/570 nm for DiI, and 350/461 nm for Hoechst 33342.

For flow cytometry experiments, HUVEC were seeded in 24-well plates. Cells were stimulated with LPS (1 μg/ml) and incubated with SAINT-O-Somes in a similar way as described for the fluorescence microscopy experiments. For competition experiments, a 50-fold excess of anti-VCAM-1 monoclonal antibodies over the amount of antibodies coupled to the SAINT-O-Somes were added to the cells together with the liposomes. 5h or 21h after addition of SAINT-O-Somes, cells were washed with PBS and detached from the surface using trypsin/EDTA (Sigma, Ayrshire, UK) after which they were immediately transferred to tubes containing PBS with 5% FCS and kept on ice. Next, samples were centrifuged for 5 min at 500 g at 4°C, followed by two wash steps with 3 ml of PBS/5% FCS and resuspended in 0.2 ml PBS for flow cytometry analysis (Calibur, BD Biosciences, Franklin Lakes, NJ, USA). When flow cytometry was performed the following day, cells were fixed with 0.5% paraformaldehyde in PBS and stored at 4°C.

### Animals

Male C57bl/6OlaHsd mice (18–23 g) were purchased from Harlan (Zeist, The Netherlands) and randomly divided into experimental groups (n = 6 per group). The animals were housed individually and maintained on a mouse chow diet in a temperature and light-dark cycle controlled environment (24°C, 12 : 12h). All intravenous (i.v.) injections were performed in the orbital plexus under general anesthesia (inhalation of isoflurane/O_2_).

To induce systemic inflammation, the mice were injected intraperitoneally with LPS in 0.9% NaCl at a dose of 1 mg/kg. Three hours later, the mice received the second dose of LPS (1 mg/kg). To compare the effects of one time versus two times LPS challenge, one group of mice was injected with a single dose of LPS (2 mg/kg, i.p.) followed by an injection of 0.9% NaCl 3h later. For the intervention groups, 2h after the first injection of LPS, the mice were administered a single dose of free dexamethasone or dexamethasone containing SAINT-O-Somes conjugated with either anti-VCAM-1 or irrelevant IgG antibody (30 μg dexamethasone / 20 g body weight). Physical appearance and behavior of the mice was regularly monitored until termination 24h after the first LPS injection. At 24h, the mice were anesthetized with isoflurane/O_2_, subsequently blood was withdrawn via cardiac puncture. Organs were harvested and immediately snap frozen on liquid nitrogen or formalin fixed for paraffin embedding later on. Frozen organs were stored at -80°C until analysis. All animal experiments were performed according to national guidelines and with approval of the local Animal Care and Use Committee of the University of Groningen (DEC6142).

### Gene expression analysis by real-time RT-PCR

Total RNA was isolated from tissue cryosections of mouse kidney, lungs, and liver using the RNeasy Mini plus Kit, (Qiagen, Westburg, Leusden, The Netherlands) according to the manufacturer’s instructions. Integrity of RNA was determined by gel electrophoresis, while RNA concentration (OD260) and purity (OD260/OD280) were measured using a NanoDrop® ND-1000 UV-Vis spectrophotometer (NanoDrop Technologies, Rockland, DE, USA). cDNA synthesis and real-time PCR were performed as described previously [18]. The Assay-on-Demand primers purchased from Applied Biosystems (Nieuwerkerk aan den IJssel, The Netherlands) for quantitative PCR included the housekeeping gene GAPDH (glyceraldehyde-3-phosphate dehydrogenase, assay ID Mm99999915_g1), CD31 (Platelet endothelial cell adhesion molecule, PECAM-1, assay ID Mm00476702_m1), VE-Cad (VE-Cadherin, assay ID Mm00486938_m1), KLF2 (Kruppel-like factor-2, assay ID Mm00500486_g1), Tie2 (receptor tyrosine kinase, assay ID Mm00443242_m1), Ang2 (Angiopoietin-2, assay ID Mm00545822_m1), E-selectin (assay ID Mm00441278_m1), VCAM-1 (vascular cell adhesion molecule-1, assay ID Mm00449197_m1), ICAM-1 (intracellular adhesion molecule-1, assay ID Mm00516023_m1), MCP1 (monocyte chemotactic protein 1, assay ID Mm00441242_m1), IL-6 (Interleukin-6, assay ID Mm00446190_m1), IL-8 (Interleukin-8, assay ID Mm00433859_m1), TNF-α (tumor necrosis factor α, assay ID Mm00443258_m1), NGAL (Neutrophil gelatinase-associated lipocalin, assay ID Mm01324470_m1), and Kim1 (Kidney injury molecule 1, assay ID Mm00506686_m1). Quantitative PCR was performed in a ViiATM 7 real-time PCR System (Applied Biosystems, Nieuwerkerk aan den IJssel, The Netherlands). Gene expression levels were normalized to the expression of the housekeeping gene GAPDH (glyceraldehyde-3-phosphate dehydrogenase). The mRNA levels relative to GAPDH were calculated by 2^-ΔCT^ values and averaged per group. The fold change of gene expression relative to the control was calculated.

### Protein quantification by enzyme-linked immunosorbent assay (ELISA)

The concentration of neutrophil gelatinase associated lipocalin (NGAL) in plasma was measured by ELISA (Quantikine®, R&D Systems, Minneapolis, MN, USA) according to the manufacturer’s instruction.

### Immunohistochemical and immunofluorescent detection of adhesion molecule protein and localization of anti-VCAM-1 dexamethasone SAINT-O-Somes in mouse tissues

Tissue cryo sections (5 μm) were fixed in acetone for 10 min. Endogenous peroxidase was blocked by 10 min incubation with Peroxidase Block (EnVision + System-HRP (AEC), DAKO, Carpentaria, CA, USA). For immunohistochemical detection of specific proteins, sections were incubated for 60 min at room temperature with primary rat anti-mouse antibodies (10 μg/ml) recognizing E-selectin (MES-1, 10 μg/ml, kindly provided by Dr. Derek Brown, UCB Celltech, Brussels, Belgium) and VCAM-1 (1: 50, clone M/K-2.7, ATCC, Manassas VA, USA) diluted in PBS/5% FCS. This was followed by 30 min incubation with unconjugated rabbit anti-rat IgG antibody (mouse adsorbed, Vector Laboratories, Burlingame, CA, USA) diluted 1: 300 in PBS/5% FCS supplemented with 2% normal mouse serum (Sanquin, Amsterdam, The Netherlands) at room temperature. Sections were then incubated for 30 min at room temperature with anti-rabbit labeled polymer HRP antibody from the EnVision kit (DAKO). Between incubations with different antibodies, sections were washed extensively with PBS. Peroxidase activity was detected with 3-amino-9-ethylcarbazole (AEC) from the EnVision kit and sections were counterstained with Mayer’s hematoxylin (Merck, Darmstadt, Germany).

Direct immunohistochemical detection of dexamethasone containing anti-VCAM-1 and IgG SAINT-O-Somes after i.v. administration was performed on the cryo sections with rabbit anti-rat antibody (Vector) in PBS/2% normal mouse serum and using EnVision kit to detect peroxidase activity as described above.

Immunofluorescence double staining to localize antibody modified SAINT-O-Somes and CD31 was performed as previously described [13]. Briefly, endogenous biotin was blocked by a Biotin Blocking System (DAKO, Glostrup, Denmark). Anti-VCAM-1 and IgG dexamethasone SAINT-O-Somes were detected with Alexa Fluor®_555_-conjugated donkey anti-rat antibody (cat.no. A-31572, Molecular Probes, Leiden, The Netherlands) diluted 1:100 in PBS/5% FCS in the presence of 2% normal mouse serum. To detect CD31, sections were incubated with biotin conjugated rat anti-mouse CD31 primary antibody (1: 200, cat. no. #550274, BD Pharmingen, San Diego, CA, USA), followed by AlexaFluor®_488_-conjugated streptavidin (1: 100, Molecular Probes) plus 2% normal mouse serum. All incubation steps were carried out in the presence of 5% FCS (Sigma-Aldrich). After proper washing, sections were then incubated with 0.1% Sudan Black B (Sigma-Aldrich) in 70% ethanol for 30 min. Slides were washed with PBS and mounted using Aqua Poly/Mount medium (Polysciences, Warrington, PA, USA) containing DAPI (4',6-Diamidino-2-Phenylindole, Dihydrochloride; Molecular Probes), air dried for 24h, and stored in the dark at 4°C. Fluorescence images were taken with a Leica DM/RXA fluorescence microscope using Quantimet HR600 image analysis software (Leica, Wetzlar, Germany).

## Results

### Ab_VCAM-1_ dexamethasone SAINT-O-Somes specifically associate with LPS-activated primary endothelial cells *in vitro*

**Fig 1:**
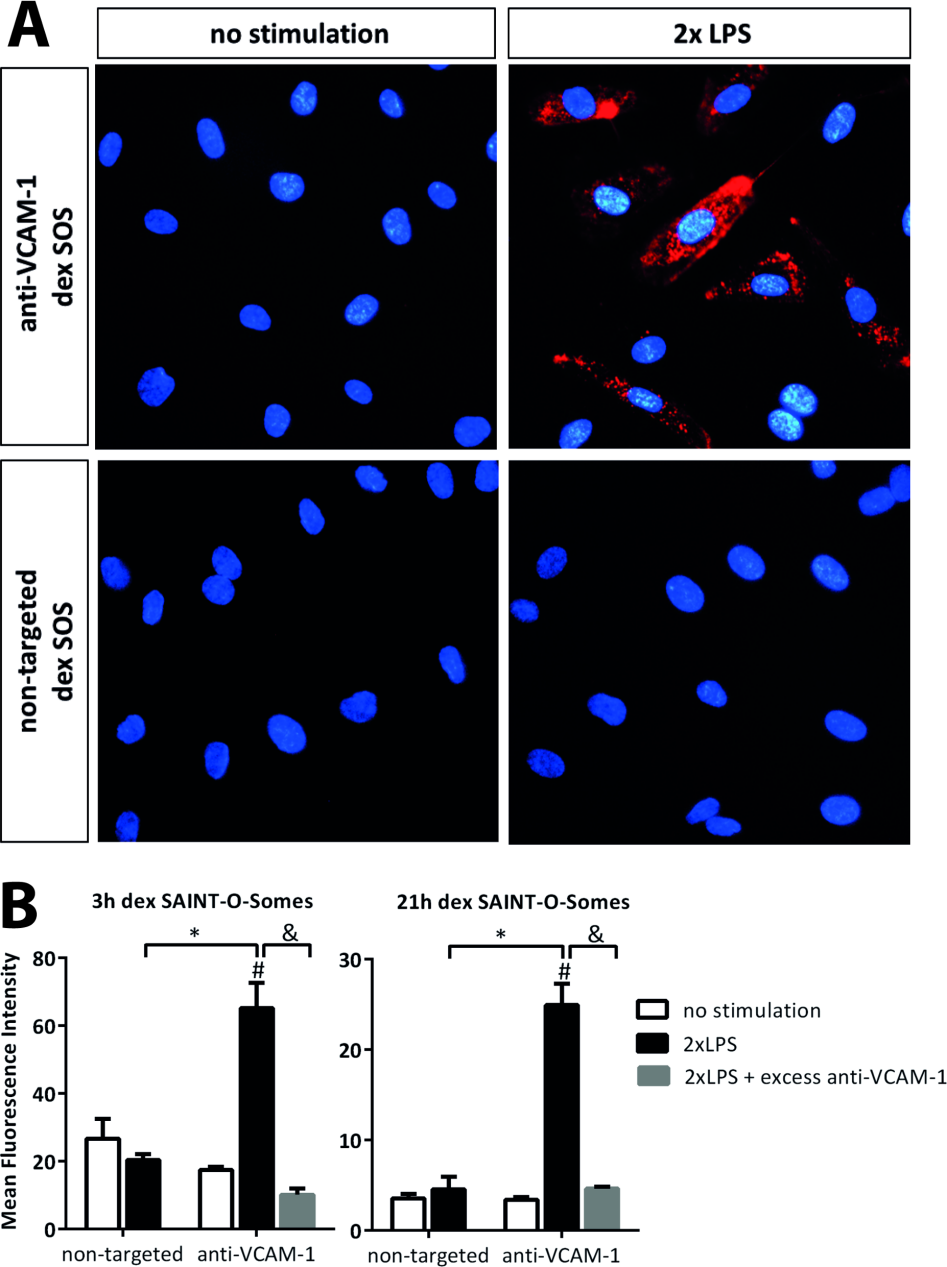
AbVCAM-1 dexamethasone SAINT-O-Somes specifically associate with LPS-activated primary endothelial cells in vitro. Human umbilical vein endothelial cells (HUVEC) were stimulated twice with LPS. The second stimulation was given 3h after the first challenge. Then quiescent respectively LPS activated HUVEC were incubated with anti-VCAM-1 and non-targeted dexamethasone containing SAINT-O-Somes (dex SOS). (A) Fluorescence microscopy images show the uptake of targeted SAINT-O-Somes by activated HUVEC after 4h incubation. The liposome membrane was labeled with Dil (red) and the nuclei of the cells were stained using Hoechst 33342 (blue). Original magnification 400x. (B) Specificity of AbVCAM-1 SAINT-O-Some association to VCAM-1 was determined by co-incubation of cells with 50 times excess of anti-VCAM-1 monoclonal antibodies together with AbVCAM-1 SAINT-O-Somes. After 3h respectively 21h co-incubation, the association of SAINT-O-Somes with activated HUVEC was quantified by flow cytometric analysis. Data are presented as mean fluorescence intensity (MFI) values ± SD of triplicate samples from one experiment. *, P < 0.05, AbVCAM-1 SAINT-O-Somes vs. non-targeted SAINT-O-Somes; #, P<0.05, association of AbVCAM-1 SAINT-O-Some with LPS stimulated HUVEC vs. unstimulated HUVEC; &, P<0.05, significant difference between with or without excess anti-VCAM-1 antibodies.

Anti-VCAM-1 SAINT-O-Somes were prepared to achieve specific delivery of dexamethasone into activated endothelial cells. LPS stimulated HUVEC were incubated with DiI labeled dexamethasone SAINT-O-Somes to determine the binding of the nanoparticles to endothelial cells. Fluorescence microscopy showed increased association of Ab_VCAM-1_ SAINT-O-Somes with LPS-activated HUVEC compared to non-activated cells (Fig 1A). In addition, SAINT-O-Somes without anti-VCAM-1 antibodies on their surface were not taken up by either resting or activated cells. To prove that the uptake of targeted SAINT-O-Somes by activated endothelial cells was VCAM-1 specific, cells were co-incubated with an excess of free antibody against the target protein VCAM-1. The significant decrease in binding of DiI labeled Ab_VCAM-1_ SAINT-O-Somes to LPS-activated HUVEC in the presence of the excess of anti-VCAM-1 antibodies confirmed the specificity of association of the nanoparticles with VCAM-1 expressed by activated HUVEC (Fig 1B).

### Two consecutive challenges of LPS result in microvascular responses in organs comparable to those after a single LPS challenge

**Fig 2:**
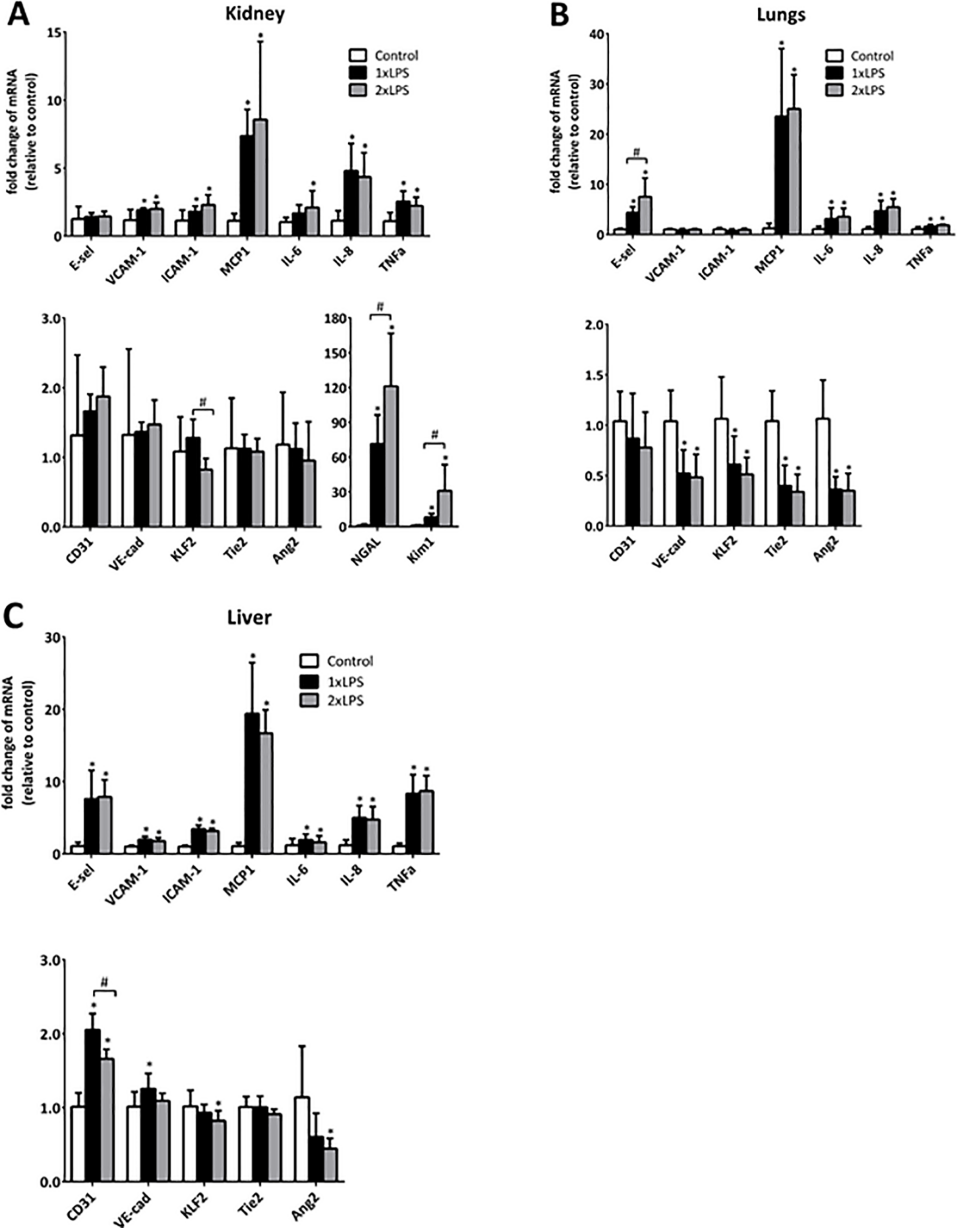
Two consecutive challenges of LPS result in microvascular responses in organs comparable to those induced by a single LPS challenge. Mice were challenged with LPS by one single i.p. injection (2mg/kg) or two consecutive injections (1mg/kg per injection), with the second injection 3h after the first challenge. 24h after the initial challenge, mRNA expression of endothelial pro-inflammatory molecules, vascular integrity related molecules, blood flow-sensitive transcription factor KLF2 in kidney (A), lungs (B), and liver (C), and kidney damage related markers NGAL and Kim1 (A) were determined by real time RT-PCR using GAPDH as housekeeping gene. All data are presented as fold change relative to saline control. Values are shown as mean ± SD (n = 6 per group). *. P<0.05, 1xLPS respectively 2xLPS vs. control; #, P<0.05, 2xLPS vs. 1xLPS.

We compared the microvascular responses of mice subjected to endotoxemia induced by a single and a double LPS challenge 24 h after the initial challenge. This time point was chosen, based on previous work [13,15,16], taken into account the kinetics of VCAM-1 expression and the pharmacokinetics of the SAINT-O-Somes we apply in this study. 24h after the initial challenge, the gene expression of pro-inflammatory adhesion molecules and cytokines in kidney, lung, and liver upon double LPS challenge was comparable with their expression induced by a single LPS injection, with a few exceptions. In Kidney, two injections of LPS lead to a lower expression of KLF2 compared to one time LPS challenge, while the increased levels of kidney damage markers NGAL and Kim1 were significantly higher upon double LPS challenge compared to their levels induced by single LPS administration. E-selectin in lungs was higher and CD31 in liver was lower after two time LPS challenge compared to single LPS injection (Fig 2). 24h after one time respectively two time LPS challenge, Tie2 mRNA expression was significantly dowregulated in lung compared to saline control, while at this time point no differences between groups of control and LPS challenged mice were observed in kidney and liver. Also, the downregulation of VE-cadherin induced by LPS challenge was restricted to lung at this time point. The expression level of Ang2 was lower both in single and double LPS challenged mice than in untreated mice in both lungs and liver at 24h. Since there was no extensive difference in the microvascular pro-inflammatory responses at 24h between the single and double LPS challenged mice, we focused our further study on the double LPS model.

### Double LPS challenge results in an abundant VCAM-1 expression and leukocyte recruitment in kidney, lung, and liver

**Fig 3:**
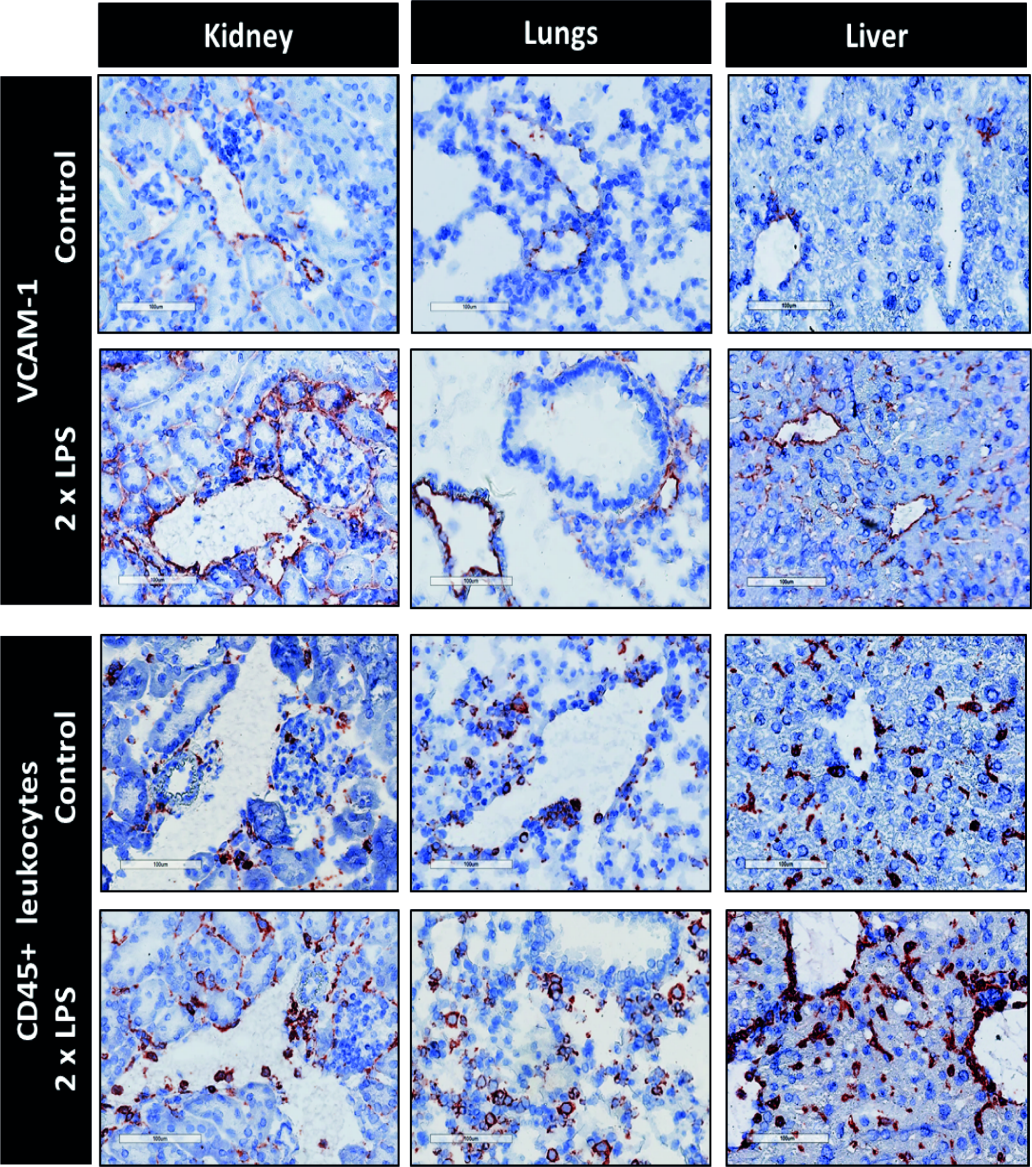
Double LPS challenge induces VCAM-1 expression and CD45+ leukocyte recruitment in organs. Immunohistochemical staining of adhesion molecule VCAM-1 and CD45+ leukocytes. Staining was performed on organ sections of control mice and mice with two consecutive LPS challenges. Mice were sacrificed 24h after the first challenge. VCAM-1 and CD45+ leukocytes stain red. Original magnification: 200x.

24h after the two consecutive LPS challenges, VCAM-1 was abundantly expressed in most of the microvascular segments of the organs investigated, i.e., arterioles, peritubular capillaries and post-capillary venules in kidney, arterioles in lung, and large venules and sinusoidal endothelium in liver (Fig 3). Furthermore, immunohistochemical staining of CD45 shows that the adhesion/infiltration of CD45+ leukocytes in the microvasculature was increased following LPS challenge.

### Ab_VCAM-1_ dexamethasone SAINT-O-Somes localization follows the expression patterns of VCAM-1 protein in the microvasculature of organs of LPS challenged mice

**Fig 4:**
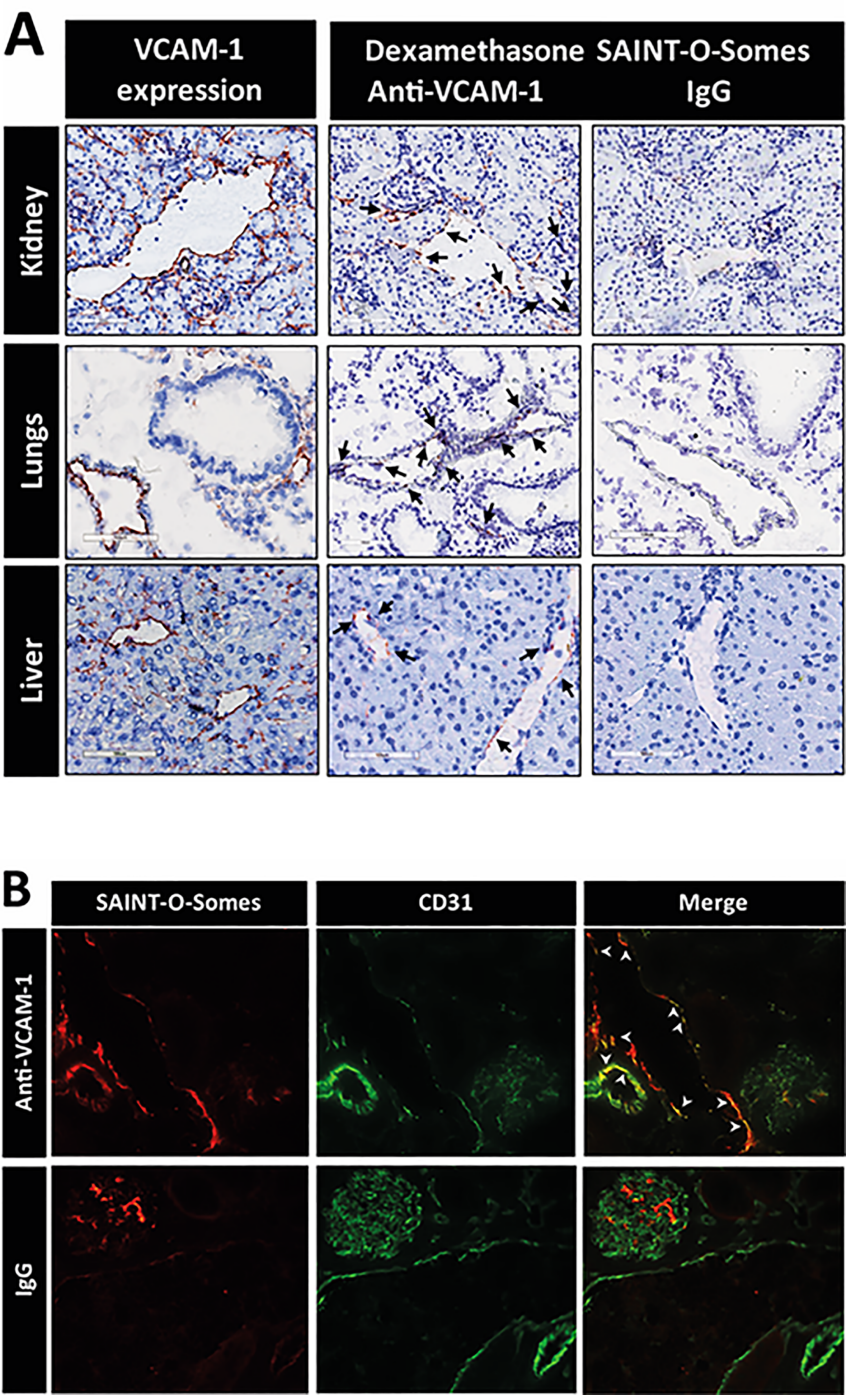
AbVCAM-1 dexamethasone SAINT-O-Somes localization follows the expression patterns of VCAM-1 protein in the microvasculature of organs of LPS challenged mice. (A) Immunohistochemical detection of VCAM-1 protein and localization of dexamethasone loaded SAINT-O-Somes liposomes in kidney, lung, and liver. LPS was administered intraperitoneally with 3 hours interval, and AbVCAM-1 respectively IgG SAINT-O-Somes administered 2h after the first LPS injection. Mice were sacrificed 24h after the first LPS injection. VCAM-1 expression was detected in double LPS challenged mice without intervention, and the localization of dexamethasone containing AbVCAM-1 respectively IgG SAINT-O-Somes was detected in intervention groups. Black arrows indicate the location of SAINT-O-Somes and liposomes in kidney, lung, and liver. Original magnification: 200x. The images are representative of 3 mice per group.

We hypothesized that in the animals exposed to LPS, the first LPS challenge allows Ab_VCAM-1_ SAINT-O-Somes to home to their target vasculature when administered to the mice 2h after the first LPS injection. Immunohistochemical staining to determine the intra-organ localization of SAINT-O-Somes indeed showed homing of Ab_VCAM-1_ SAINT-O-Somes. Though less intense the staining of the Ab_VCAM-1_ SAINT-O-Somes followed the expression patterns of VCAM-1 in kidney, lungs, and liver as indicated by the arrows (Fig 4A). Ab_VCAM-1_ SAINT-O-Somes were visible in arterioles, venules and peritubular capillaries in the kidney, arterioles in the lung, and large venules in the liver. Non-targeted IgG SAINT-O-Somes were found to slightly accumulate in the glomerular compartment of the kidney while being undetectable in the other renal microvasculatures as well as in lungs and liver. The association of Ab_VCAM-1_ SAINT-O-Somes with endothelial cells in the kidney was confirmed by immunofluorescence double staining showing co-localization of Ab_VCAM-1_ SAINT-O-Somes with CD31 mainly in the venules and arterioles of LPS challenged mice (Fig 4B). The localization of Ab_VCAM-1_ SAINT-O-Somes indicates association of the dexamethasone containing nanoparticles with VCAM-1 expressed on endothelial cells in specific microvascular segments in mice challenged with LPS.

Fluorescence microscopy images of immunofluorescent staining for endothelial marker CD31 (green) and anti-VCAM-1 dexamethasone SAINT-O-Somes (red) (B) in kidney of LPS double LPS challenged mice 24h after the first LPS challenge. White arrowheads point at arterioles and venules and indicate the merged staining of CD31 and SAINT-O-Somes. Original magnification: 400x. The images are representative of 2 mice per group.

### Effects of endothelial targeted Ab_VCAM-1_ dexamethasone SAINT-O-Somes in organs of double LPS challenged mice

**Fig 5:**
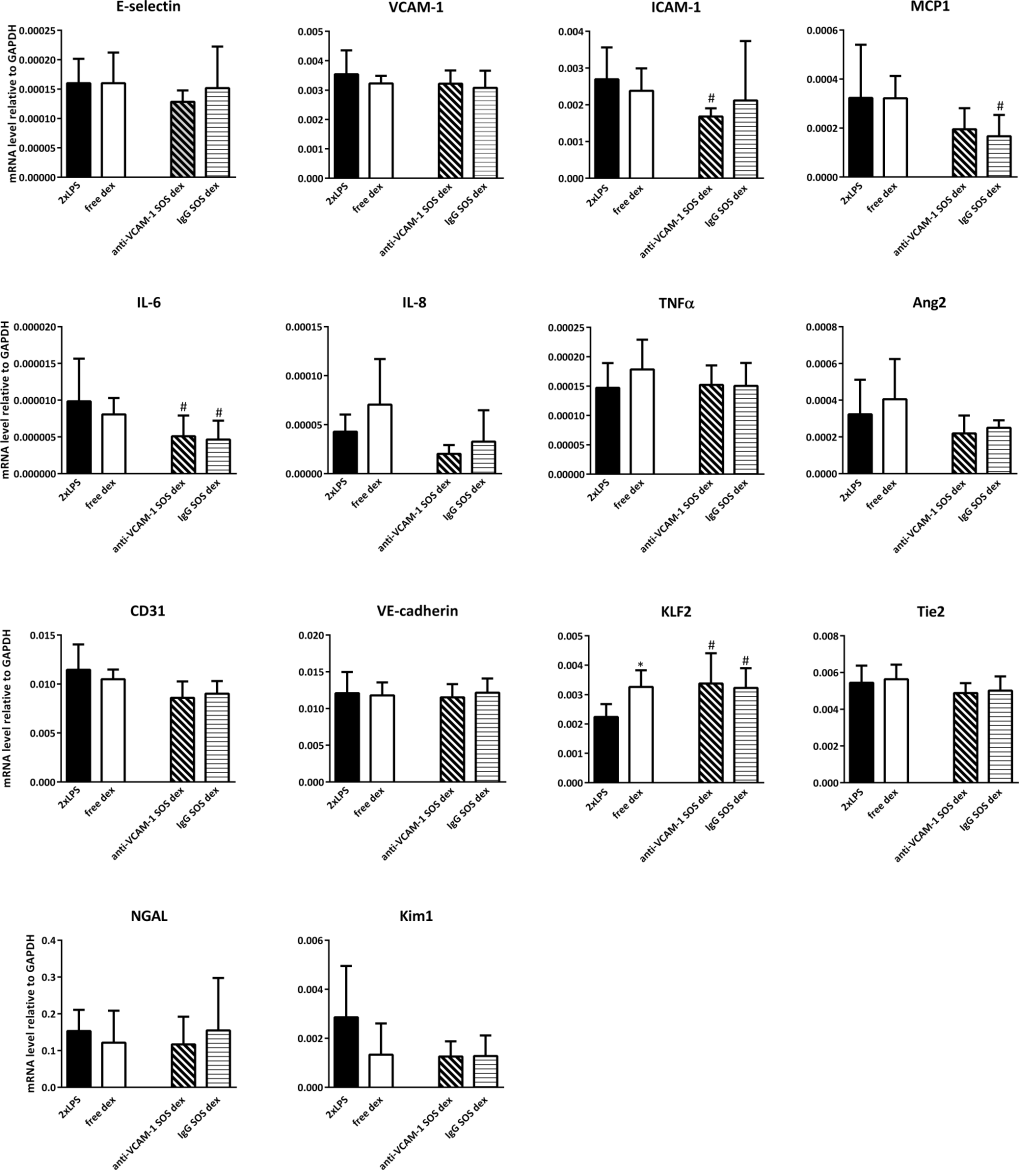
Effects of endothelial targeted AbVCAM-1 dexamethasone SAINT-O-Somes in kidney of double LPS challenged mice. mRNA expression of endothelial pro-inflammatory molecules, vascular integrity related molecules, blood flow-sensitive transcription factor KLF2, and organ damage related markers NGAL and Kim1 in kidney was determined by real time RT-PCR using GAPDH as a housekeeping gene. All data are presented as mRNA level relative to GAPDH. Values are shown as mean ± SD (n = 6 per group). *, P<0.05, free dexamethasone vs. 2xLPS; #, P<0.05, anti-VCAM-1 respectively IgG dexamethasone SAINT-O-Somes vs. 2xLPS.

**Fig 6:**
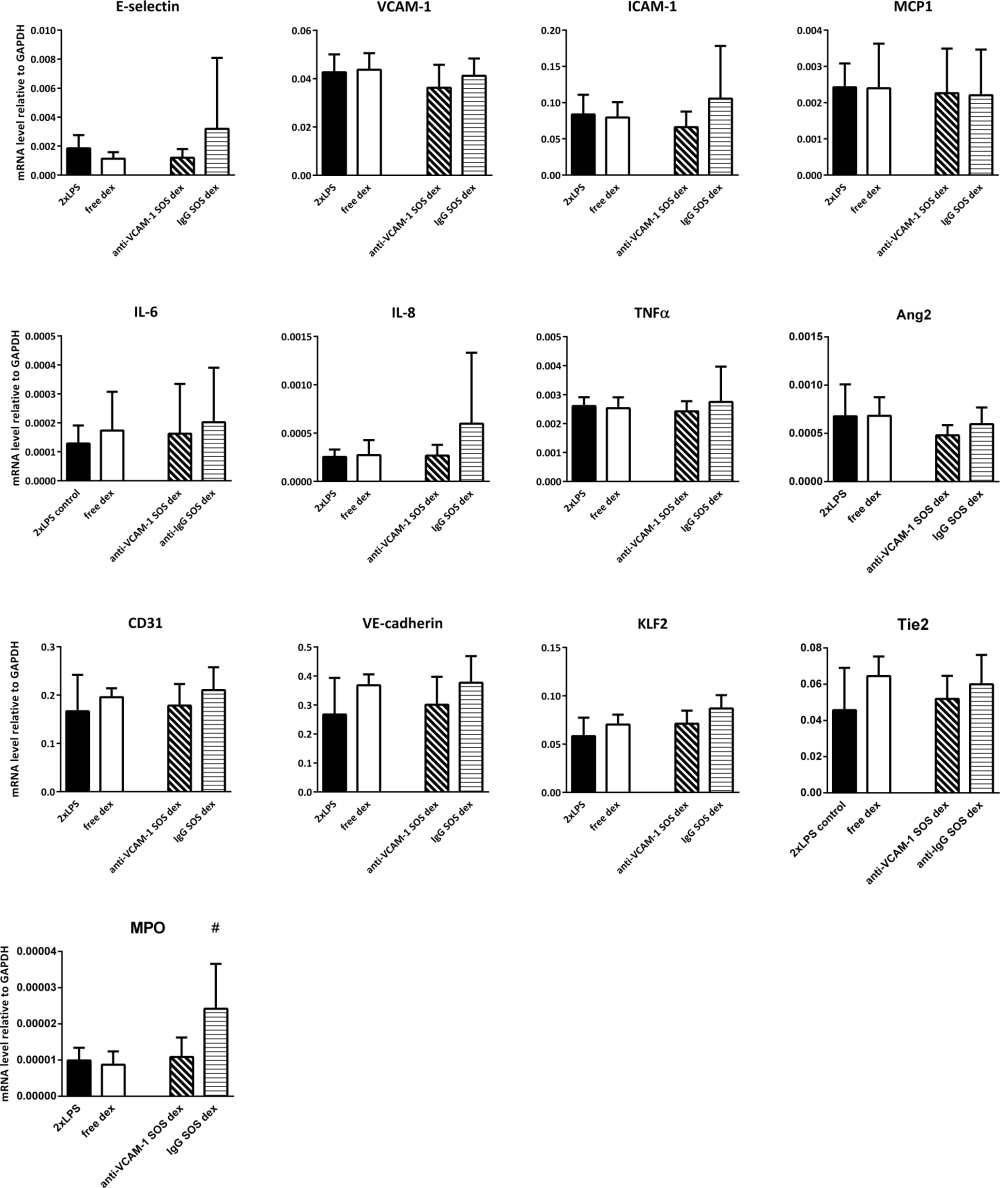
Effects of the endothelial targeted AbVCAM-1 dexamethasone SAINT-O-Somes in lungs of double LPS challenged mice. mRNA expression of pro-inflammatory molecules, vascular integrity related molecules, and blood flow-sensitive transcription factor KLF2 in lung was determined by real time RT-PCR using GAPDH as housekeeping gene. All data are presented as mRNA level relative to GAPDH. Values are shown as mean ± SD (n = 6 per group). *, P<0.05, free dexamethasone vs. 2xLPS; #, P<0.05, anti-VCAM-1 respectively IgG dexamethasone SAINT-O-Somes vs. 2xLPS.

**Fig 7:**
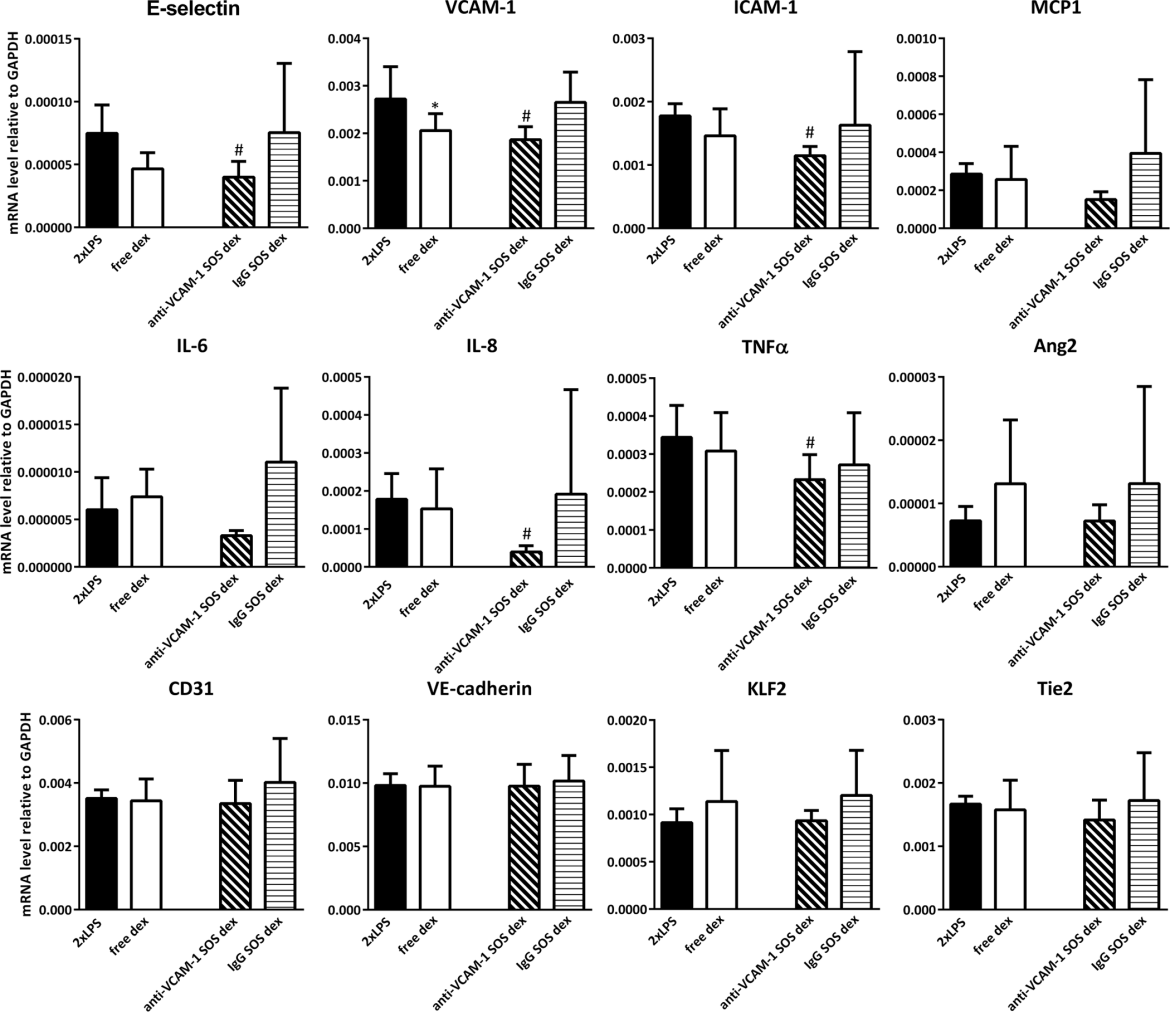
Effects of the endothelial targeted AbVCAM-1 dexamethasone SAINT-O-Somes in liver of double LPS challenged mice. mRNA expression of pro-inflammatory molecules (cell adhesion molecules and cytokines), vascular integrity related molecules (Tie2, CD31, VE-cadherin, Ang2), blood flow-sensitive transcription factor KLF2 in the liver was determined by real time RT-PCR using GAPDH as housekeeping gene. All data are presented as mRNA level relative to GAPDH. Values are shown as mean ± SD of each group (n = 6). *, P<0.05, free dexamethasone vs. 2xLPS; #, P<0.05, anti-VCAM-1 respectively IgG dexamethasone SAINT-O-Somes vs. 2xLPS.

To determine the pharmacological effects of endothelial cell targeted dexamethasone, we analyzed the expression of inflammation markers and vascular integrity related genes in kidney, lungs, and liver of double LPS challenged mice. The administration of free dexamethasone did not significantly alter their expression in these organs except for the slight upregulation of KLF2 in the kidney and a minor inhibitory effect on VCAM-1 expression in the liver (Figs 5–7). When Ab_VCAM-1_ dexamethasone SAINT-O-Somes were administered, a few endothelial adhesion molecules and pro-inflammatory cytokines were modestly reduced. In the kidney of LPS challenged mice, the expression of ICAM-1 and IL-6 was downregulated and KLF2 was upregulated (Fig 5) while no effect was found in the lung (Fig 6). In the liver, Ab_VCAM-1_ dexamethasone SAINT-O-Somes administration significantly decreased the expression of E-selectin, VCAM-1, ICAM-1, IL-8, and TNFα (Fig 7). Non-targeted IgG dexamethasone SAINT-O-Somes showed reduction of MCP1 and induction of KLF2 in the kidney while no effect was observed in lungs and liver (Figs 5–7).

## Discussion

The microvascular endothelium plays a pivotal role in regulating leukocyte recruitment, hypotension and vascular leakage associated with sepsis. Therefore, endothelial cell directed therapeutic interventions are a promising approach for sepsis treatment [2]. In the present study mice were challenged with two doses of LPS to induce sepsis associated microvascular endothelial cell activation. Comparison of one dose of LPS with two consecutive doses of LPS did not reveal major differences. Since the double-hit model might mimic the clinical sepsis closer than the one dose model, the dexamethasone intervention study was performed in the double-hit model. We investigated the effectiveness of free dexamethasone and dexamethasone loaded immune SAINT-O-Somes targeted to VCAM-1 expressed endothelial cells in inhibiting LPS mediated microvascular endothelial activation. We showed that the administration of free dexamethasone hardly altered microvascular endothelial activation. Ab_VCAM-1_ dexamethasone SAINT-O-Somes selectively homed to VCAM-1 expressed endothelial cells in dedicated microvascular segments in organs of LPS challenged mice. The pharmacological effects of inhibiting microvascular endothelial activation by Ab_VCAM-1_ dexamethasone SAINT-O-Somes were marginal.

The moderate effects of free and endothelial cell targeted dexamethasone in LPS challenged mice to reduce microvascular endothelial activation as shown in this study might help to explain why non-targeted corticosteroids are not effective in reducing sepsis induced multiple organ failure [8]. Annane et al have reported improved survival and reversal of shock in patients who received a 7-day treatment with low doses of hydrocortisone and fludrocortisone [19]. In contrast, Sprung et al have demonstrated that hydrocortisone had no significant effect on the rate of death in patients with septic shock at 28 days [20]. Several animal studies of sepsis models have suggested that endothelial cell-restricted inhibition of endothelial inflammatory signaling pathways such as NF-κB is beneficial while its inhibition in immune cells is detrimental to the host [10]. The effect of dexamethasone targeted to endothelial cells *in vivo* is limited, suggesting that dexamethasone (GC) lacks therapeutic potential to mitigate endothelial cell activation in sepsis. Possibly, Ab_VCAM-1_ SAINT-O-Somes with other drugs as cargo can be further developed to attenuate endothelial inflammatory responses in sepsis [16].

Endothelial cell directed liposomes modified with ligands for specific receptors might improve therapeutic outcomes by increasing the concentration of the drugs at the target tissue in the target cells and simultaneously preventing healthy tissue from drug exposure, thereby reducing toxicity [12]. Endothelial cells are not equipped to process conventional liposomes, leading to inferior pharmacological effects caused by limited intracellular release of the delivered liposomal content. We previously showed that SAINT-O-Somes, generated by incorporating the cationic pyridinium-derived lipid—SAINT into the lipid bilayer of long circulating liposomes, have more efficient intracellular content release properties in endothelial cells [14]. VCAM-1 is an inducible endothelial cell adhesion molecule that is scarcely expressed in resting endothelium and highly upregulated upon inflammatory stimulation during hemorrhagic shock and sepsis [3,21]. In the present study, after LPS administration, VCAM-1 was substantially induced in all vascular segments, except in glomeruli of the kidney, the latter corroborating a previous report from our group showing that in glomerular endothelial cells higher micro-RNA-126 expression was associated with lower VCAM-1 protein translation [22]. Ab_VCAM-1_ SAINT-O-Somes consequently homed to arterioles and venules, while non-targeted IgG SAINT-O-Somes preferentially accumulated in glomeruli, which is likely a result of increased leakage of the glomerular capillaries due to LPS challenge [23]. This data shows that selective delivery of therapeutic molecules to endothelial cells in sepsis can be achieved using Ab_VCAM-1_ SAINT-O-Somes. Recently we demonstrated that endothelial cell targeted SAINT-O-Somes containing a different drug are actually capable of abrogating the disease process in ANCA-induced glomerulonephritis in mice, confirming effective homing of the SAINT-O-Somes [24].

In summary, we here reported the accumulation of dexamethasone loaded anti-VCAM-1 SAINT-O-Somes in endotoxemia activated VCAM-1 expressed microvasculature. The administration of free dexamethasone and Ab_VCAM-1_ dexamethasone SAINT-O-Somes had a marginal effect on inhibiting microvascular endothelial activation. Future studies targeting the endothelium with nano drug carriers carrying more potent and more precise drugs need to be performed to elucidate their therapeutic benefit in the treatment of sepsis.
